# Association between increased signal intensity at the proximal patellar tendon and patellofemoral geometry in community-based asymptomatic middle-aged adults: a cross-sectional study

**DOI:** 10.1186/s12891-020-03589-4

**Published:** 2020-08-22

**Authors:** Robert D. Little, Samuel E. Smith, Flavia M. Cicuttini, Stephanie K. Tanamas, Anita E. Wluka, Sultana Monira Hussain, Donna M. Urquhart, Graeme Jones, Yuanyuan Wang

**Affiliations:** 1 School of Public Health and Preventive Medicine, Monash University, 553 St Kilda Road, Melbourne, VIC 3004 Australia; 2 Institute of Vector-Borne Disease, Monash University, Clayton, VIC 3800 Australia; 3 Menzies Institute for Medical Research, University of Tasmania, Hobart, TAS 7000 Australia

**Keywords:** Patellar tendon, Insall-Salvati ratio, Patellofemoral congruence angle, Magnetic resonance imaging

## Abstract

**Background:**

Histological and epidemiological data suggest that increased signal intensity at the proximal patellar tendon on magnetic resonance imaging is a response to tendon loading. As patellofemoral geometry is a mediator of loading, we examined the association between patellofemoral geometry and the prevalence of increased signal intensity at the patellar tendon in community-based middle-aged adults.

**Methods:**

Two hundred-one adults aged 25–60 years in a study of obesity and musculoskeletal health had the patellar tendon assessed from magnetic resonance imaging. Increased signal intensity at the proximal patellar tendon was defined as hyper-intense regions of characteristic pattern, size and distribution on both T1- and T2-weighted sequences. Indices of patellofemoral geometry, including Insall-Salvati ratio, patellofemoral congruence angle, sulcus angle, and lateral condyle-patella angle, were measured from magnetic resonance imaging using validated methods. Binary logistic regression was used to examine the association between patellofemoral geometrical indices and the prevalence of increased signal intensity at the patellar tendon.

**Results:**

The prevalence of increased signal intensity at the patellar tendon was 37.3%. A greater Insall-Salvati ratio (odds ratio 0.80, 95% confidence interval 0.66–0.97 per 0.1 change in the ratio, *p* = 0.02), indicative of a higher-riding patella, and a larger patellofemoral congruence angle (odds ratio 0.91, 95% confidence interval 0.85–0.98 per 5 degree change in the angle, *p* = 0.01), indicating a more laterally placed patella, were associated with reduced odds of increased signal intensity at the patellar tendon. Sulcus angle and lateral condyle-patella angle were not significantly associated with the odds of increased signal intensity at the patellar tendon.

**Conclusions:**

In community-based asymptomatic middle-aged adults, increased signal intensity at the patellar tendon was common and associated with Insall-Salvati ratio and patellofemoral congruence angle, suggesting a biomechanical mechanism. Such work is likely to inform tissue engineering and cell regeneration approaches to improving outcomes in those with tendon pathology.

## Background

The structure-function relationships in articular cartilage and subchondral bone have been widely investigated, but relatively little attention has been given to tendons. The increasing interest and effort in cell regeneration therapy and tissue engineering to promote tendon repair and create artificial tendons [1, 2] highlight the need for such work to be underpinned by a comprehensive understanding of the functional morphology of tendons and the risk factors for tendon abnormalities.

Magnetic resonance imaging (MRI) enables non-invasive assessment of joint structures with signal alterations reflecting tissue composition. Histological examination of increased signal intensity of the proximal patellar tendon on T1-weighted and fluid-sensitive MRI have been shown to be due to invaginated adipose tissue, vessels, and perivascular connective tissue [3]. We have shown increased signal intensity at the proximal patellar tendon to be common in otherwise asymptomatic community-based adults aged over 40 years and to be associated with obesity, high levels of physical activity and increased size of vastus medialis, suggesting a predominant biomechanical mechanism [4, 5]. These findings, together with the histological changes within the region of increased signal intensity at the proximal patellar tendon, support a tissue response to increased loading in the patellar tendon.

Patellofemoral geometry has an important role on the direction and magnitude of forces acting on the patellofemoral joint and patellofemoral alignment may affect load distribution to the extensor mechanism, thus altering the distribution of strain within the patellar tendon upon quadriceps contraction [6, 7]. Given the potential biomechanical mechanism for the presence of increased signal intensity at the patellar tendon [4, 5], we examined the association between patellofemoral geometry and the prevalence of increased signal intensity at the proximal patellar tendon in community-based asymptomatic middle-aged individuals. We hypothesised that altered patellofemoral geometry would be associated with an increased odds of increased signal intensity at the proximal patellar tendon.

## Methods

### Study participants

Two hundred and fifty participants, aged 25–60 years and across the spectrum from normal weight to obese, were recruited into a study investigating the effect of obesity and weight loss on knee health. They were recruited via advertising in the local media, hospital waiting rooms, community weight loss organisations, and private weight loss and obesity clinics [8]. Participants were excluded if they had a history of any joint disease diagnosed by a medical practitioner, significant knee injury requiring non-weight-bearing therapy, previous knee surgery including arthroscopy, knee pain requiring prescribed analgesia or no weight-bearing activity for > 24 h, contraindication to MRI, or malignancy. The Monash University Human Research Ethics Committee and the Alfred Hospital Ethics Committee approved this study. Written informed consent was obtained from all the participants.

### Assessment of anthropometry and physical activity

Weight was measured using a single pair of electronic scales to the nearest 0.1 kg (shoes and bulky clothing removed). Height was measured using a stadiometer to the nearest 0.1 cm (shoes removed). Body mass index (BMI) was calculated (kg/m^2^). Strenuous physical activity was assessed as previously described [9] by asking “On how many days during the last 14 days did you spend at least 20 minutes doing strenuous exercise that was severe enough to raise your pulse rate, cause you to breathe faster (e.g. bicycling, brisk walking, jogging and aerobics)”. The participants were asked to select one from the five frequency options: no days; 1–2 days; 3–5 days; 6–8 days; and > 9 days. Individuals were classified as performing strenuous physical activity if they participated in strenuous activity for > 3 days during the previous 14 days.

### Acquisition of MRI

Participants underwent an MRI of their dominant knee (the lower limb from which the individual steps off from when starting to walk) on a 1.5-T whole body magnetic resonance unit (Philips, Medical Systems, Eindhoven, the Netherlands) using a commercial transmit-receive extremity coil, with two sequences obtained: (1) T1-weighted, fat-saturated 3D gradient recall acquisition in the steady state, repetition time 58 ms, echo time 12 ms, flip angle 55 degrees, field of view 16 cm, 60 partitions, 512 × 512 matrix, one acquisition. Sagittal images had a partition thickness of 1.5 mm and an in-plane resolution of 0.31 × 0.31 mm. (2) Coronal T2-weighted, fat-saturated, fast spin echo acquisition, repetition time 2200 ms, echo time 20/80 ms, flip angle 90 degrees, slice thickness 3 mm with a 0.3 inter-slice gap, 1 excitation, field of view 13 cm, and 256 × 192 matrix. MRI measurements were undertaken by independent observers who had at least two years’ experience in MRI measurement and were trained by a radiologist and blinded to participant characteristics.

### Assessment of increased signal intensity at the proximal patellar tendon

**Fig. 1:**
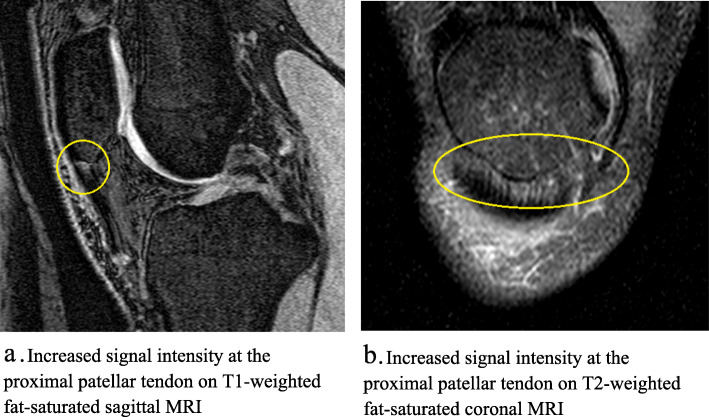
Increased signal intensity at the proximal patellar tendon on **a**. T1-weighted fat-saturated sagittal MRI; and **b**. T2-weighted fat-saturated coronal MRI

Increased signal intensity was assessed as present or absence at the proximal patellar tendon. The presence of increased signal intensity at the proximal patellar tendon was defined as a hyper-intense area with characteristic distribution, size, and pattern present on at least two contiguous slices in the proximal region of the inferior patellar tendon. This was assessed on sagittal T1-weighted fat-saturated images and then confirmed on coronal T2-weighted fat-saturated images in order to reduce the false positives due to the ‘magic angle effect’ [10] (Fig. 1). The reproducibility of the measurement was assessed using 50 randomly selected MRIs, and the intra- and inter-observer reproducibility (Cohen’s kappa) was 0.90 and 0.82, respectively.

### Measurement of patellofemoral geometry

**Fig. 2:**
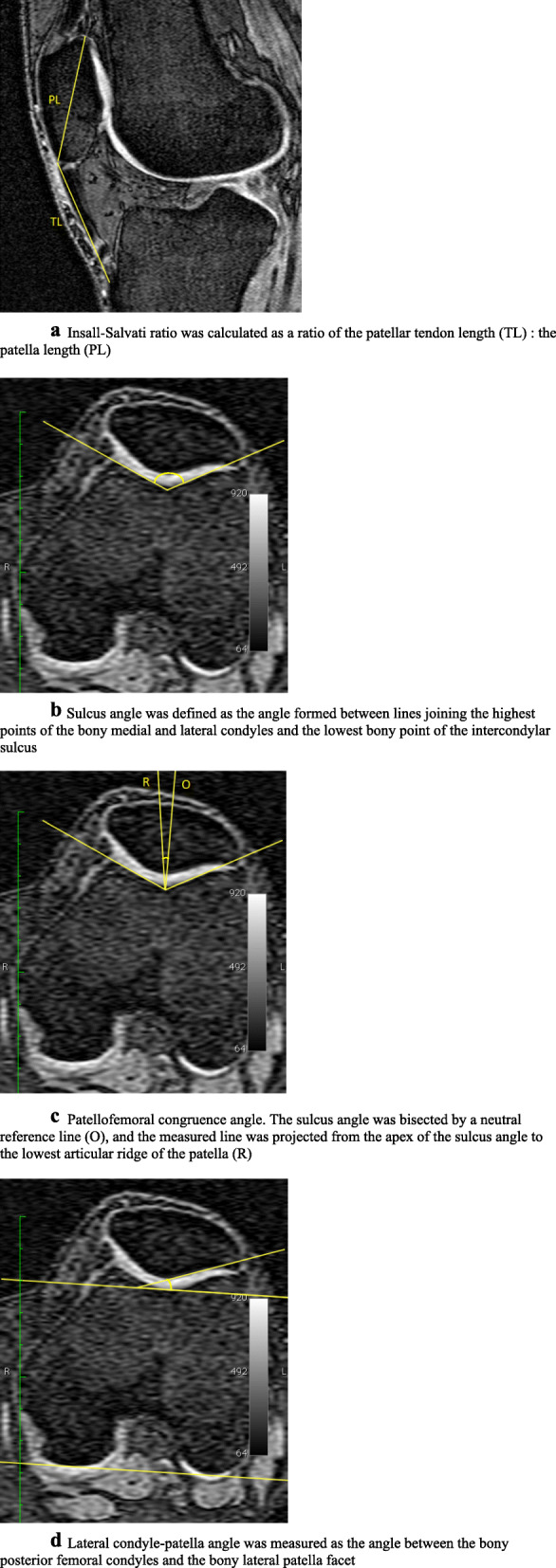
Measurement of patellofemoral geometry

Insall-Salvati ratio, sulcus angle, patellofemoral congruence angle, and lateral condyle-patella angle, were measured by image processing using the Osiris software (University of Geneva, Switzerland). Insall-Salvati ratio measures patellar height, which represents the ratio of the length of the patellar tendon to the length of the patella, measured from the sagittal plane at the midpoint of the patella (Fig. 2 a). A ratio > 1.2 corresponds to a high-riding patella and a ratio < 0.8 indicates a low-riding patella [11]. Our intra-observer reproducibility [intra-class correlation coefficient (ICC)] was 0.86 [8]. Sagittal MR images were reformatted to axial images with a resolution of 0.31 × 0.31 mm. The femoral sulcus angle was formed by lines connecting the lowest point of the intercondylar sulcus and the highest points of the bony medial and lateral femoral condyles (Fig. 2 b). Larger angles correspond to a shallower articular surface and smaller angles correspond to a deeper articular surface. Our intra-observer reproducibility (ICC) was 0.98 [8]. The patellofemoral congruence angle is an index of patellar subluxation defining the relationship between the patellar articular ridge and the intercondylar sulcus [12]. The sulcus angle was bisected by a neutral reference line, and the measured line was projected from the apex of the sulcus angle to the lowest articular ridge of the patella. A positive value was assigned if the measured line fell lateral to the reference line (Fig. 2 c), with angles above 16 degrees of lateral subluxation corresponding to abnormal lateral patella position. Our intra-observer reproducibility (ICC) was 0.99. The lateral condyle-patella angle was measured as the angle between the bony posterior femoral condyles and the bony lateral patella facet (Fig. 2 d), with larger angles indicating more medial patellar inclination and smaller angles indicating more lateral patellar inclination. Our intra-observer reproducibility (ICC) was 0.98 [8]. The three angles described above were all measured in degrees at the mid-patella level.

### Statistical analysis

The characteristics of participants with and without increased signal intensity at the patellar tendon were compared using independent samples t-test or chi square test when appropriate. Binary logistic regression was used to examine the relationship between patellofemoral geometrical indices (independent variable) and the prevalence of increased signal intensity at the patellar tendon (dependent variable), with multivariable analyses adjusted for age, gender and BMI. Patellofemoral geometry variables were entered in the regression models as continuous variables with some conversions: Insall-Salvati ratio was entered as per 0.1 (i.e raw data multiplied by 10), patellofemoral congruence angle, sulcus angle, and lateral condyle-patella angle were entered as per 5 degrees (i.e. raw data divided by 5). A *p*-value of less than 0.05 (two-tailed) was considered statistically significant. All analyses were performed using the IBM SPSS statistical package (standard version 24, SPSS, Chicago, IL, USA).

## Results

Among the 250 participants, increased signal intensity at the patellar tendon could be assessed in 201 (80.4%) participants, with 49 participants without the assessment due to the unavailability of particular T2-weighted coronal images to confirm the presence of increased signal intensity at the patellar tendon. These 49 participants had higher BMI [37.8 (SD 9.7) vs. 33.2 (SD 9.3) kg/m^2^, *p* = 0.003] and greater Insall-Salvati ratio [1.06 (SD 0.14) vs. 0.98 (SD 0.16), *p* = 0.01] compared with those with patellar tendon assessment.

Participants with increased signal intensity at the patellar tendon (*n* = 75, 37.3%) were more likely to be male (33% vs. 20%, *p* = 0.03), had lower Insall-Salvati ratio (0.94 vs. 1.00, *p* = 0.02) and smaller patellofemoral congruence angle (10.3 vs. 17.6 degrees, *p* = 0.04), compared with those without increased signal intensity at the patellar tendon (Table 1). No significant differences were found for age, BMI, sulcus angle, or lateral condyle-patella angle between the two groups.

**Table 1:** Characteristics of study participants

	No increased signal intensity at patellar tendon (*n* = 126)	Increased signal intensity at patellar tendon (*n* = 75)	*P*
Age, years	46.3 (9.3)	46.3 (9.7)	0.98
Female, n (%)	101 (80.2)	50 (66.7)	0.03
Body mass index, kg/m^2^	33.6 (9.5)	32.6 (9.1)	0.49
Strenuous physical activity, n (%)	100 (79.4)	59 (78.7)	0.91
**Patellofemoral geometrical indices**			
Insall-Salvati ratio	1.00 (0.16)	0.94 (0.16)	0.02
Patellofemoral congruence angle, degrees	17.6 (24.4)	10.3 (23.2)	0.04
Sulcus angle, degrees	150.9 (10.1)	149.9 (9.5)	0.48
Lateral condyle-patella angle, degrees	19.4 (7.2)	19.1 (6.6)	0.81

Data presented as mean (SD) or no (%)

In univariable analyses, a higher Insall-Salvati ratio and a greater patellofemoral congruence angle were associated with reduced odds of increased signal intensity at the patellar tendon (Table 2). After adjustment for age, gender and BMI, a greater Insall-Salvati ratio [odds ratio 0.80, 95% confidence interval 0.66–0.97, *p* = 0.02] and a larger patellofemoral congruence angle (odds ratio 0.91, 95% confidence interval 0.85–0.98, *p* = 0.01) remained significantly associated with decreased odds of increased signal intensity at the patellar tendon. There was no significant association for sulcus angle or lateral condyle-patella angle in either univariable or multivariable analysis (Table 2). Adding strenuous physical activity in the regression models did not change the results (data not shown).

**Table 2:** Association between patellofemoral geometrical indices and the prevalence of increased signal intensity at the patellar tendon

	Univariable analysis Odds ratio (95% CI)	*P* value	Multivariable analysis* Odds ratio (95% CI)	*P* value
Insall-Salvati ratio (per 0.1)	0.80 (0.66, 0.97)	0.02	0.80 (0.66, 0.97)	0.02
Patellofemoral congruence angle (per 5 degrees)	0.94 (0.88, 1.00)	0.04	0.91 (0.85, 0.98)	0.01
Sulcus angle (per 5 degrees)	0.95 (0.82, 1.10)	0.48	0.95 (0.82, 1.11)	0.52
Lateral condyle-patella angle (per 5 degrees)	0.98 (0.79, 1.20)	0.81	0.98 (0.79, 1.21)	0.82

*CI* confidence interval

*Adjusted for age, gender, and body mass index

## Discussion

This study showed that increased signal intensity at the proximal patellar tendon on T1-weight and fluid-sensitive MRI was present in over one third of community-based, asymptomatic middle-aged individuals. A greater Insall-Salvati ratio, indicating a high-riding patella, and a larger patellofemoral congruence angle, reflecting a more laterally placed patella (subluxation), were associated with reduced odds of increased signal intensity at the proximal patellar tendon.

The high prevalence of increased signal intensity at the proximal patellar tendon in our study provides an opportunity to understand patellar tendon health given that this MRI change is known to represent distinct pathological processes of invaginated adipose tissue, vessels, and perivascular connective tissue [3]. A high-riding patella and a laterally placed patella have been associated with a range of patellofemoral disorders including radiographic patellofemoral osteoarthritis [13], MRI-derived structural features of patellofemoral osteoarthritis (such as bone marrow lesions and cartilage damages) and their worsening [14–16], and recurrent patellar dislocation [17]. Our study found reduced odds of increased signal intensity at the proximal patellar tendon in relation to a greater Insall-Salvati ratio in middle-aged, asymptomatic, predominantly obese adults. Redistribution of mechanical load would be one potential mechanism to explain our result, as it has been suggested that a high-riding patella may increase antero-posterior load bearing through the patellar cartilage, which in turn offloads patellar tendon stress, leading to reduced connective tissue damage and less signal abnormality within the patellar tendon.

The reduced odds of increased signal intensity at the patellar tendon related to a larger patellofemoral congruence angle observed in our study is supported by our previous study reporting an association between greater vastus medialis cross-sectional area and higher prevalence of increased signal intensity at the patellar tendon [5]. Greater vastus medialis activity reduces lateral patella stress via increased medial tension [18], and greater vastus medialis size may therefore reduce lateral patella maltracking and patellofemoral congruence angle which diminishes articular cartilage stress and thereby ameliorates any reduction in patellar tendon load. The relationship observed in our study between increased signal intensity at the proximal patellar tendon and Insall-Salvati ratio and patellofemoral congruence angle suggests a biomechanical mechanism for its pathogenesis. Altering patellofemoral joint mechanics by targeting patellofemoral geometry may provide a potential approach to improving patellar tendon morphology and treatment outcomes for people with tendon pathology.

Limitations of our study include the cross-sectional analyses for the association between patellofemoral geometry and increased signal intensity at the proximal patellar tendon, and thus the causality cannot be examined. While highly reproducible, the increased signal intensity at the proximal patellar tendon detected from MRI may not correlate with clinical disease [19, 20]. However, longitudinal studies have demonstrated that abnormalities within asymptomatic tendon identified by ultrasound predicted the subsequent development of tendon-related symptoms and disability [21, 22]. Therefore, the increased signal intensity at the patellar tendon in asymptomatic participants may be demonstrating the early phase of structural changes in the patellar tendon in reaction to biomechanical factors. In this study femoral sulcus angle, patellofemoral congruence angle, and lateral condyle-patella angle were measured on axial images. Although there was some loss of information when reformatting the original sagittal images to the axial images, the axial images had a good resolution of 0.31 × 0.31 mm. All the angle measurements were based on the anatomical landmarks of bone and cartilage which were clearly shown on the axial images. Furthermore, all the images were processed in the same way and all the angle measurements were performed in the same way for all the participants with high reproducibility. Therefore the reformatting of images is unlikely to have significantly affected the angle measurements or the results of the study. Participants in our study underwent non-weight-bearing geometrical measurements of the patellofemoral joint with the knee in full extension which is a suboptimal representation of joint motion and weight-bearing in day-to-day activity [23]. Traditionally, patellofemoral geometry was first measured on plain radiographs. Insall-Salvati ratio was measured on lateral radiographs [11], and femoral sulcus angle [24], patellofemoral congruence angle [12], and lateral condyle-patella angle [25] were measured on skyline view radiographs, all with patients in a non-weight-bearing position. Although the measurement of patellofemoral alignment would be expected to be affected by the patient position (supine, prone, weight-bearing), the evidence is inconclusive regarding the potential difference in patellofemoral alignment between the weight-bearing and non-weight-bearing conditions [26]. In this study all participants were assessed non-weight-bearing. While the absolute values of patellofemoral geometry measures might be different based on weight-bearing or not, this is unlikely to have affected our findings of the association of greater Insall-Salvati ratio and patellofemoral congruence angle with reduced odds of increased signal intensity at the patellar tendon as all participants were measured in the same way and any measurement error is likely to be non-differential which would result in an underestimation of the magnitude of observed associations. Insall-Salvati ratio is susceptible to measurement errors arising from variations in patellar position and morphology [27, 28]. The strengths of our study include the enrollment of community-based participants across a wide range of BMI, thereby increasing the generalizability of our findings. In addition, we used a non-invasive, MRI-based assessment of the patellar tendon using both T1-weighted and fluid-sensitive sequences to reduce the risk of erroneously misclassifying ‘magic angle’ signal intensity as true structural alterations in the patellar tendon.

## Conclusions

Our study showed a high prevalence of MRI-detected increased signal intensity at the proximal patellar tendon in a sample of community-based middle-aged asymptomatic adults across a range of BMI. The lower odds of increased signal intensity at the patellar tendon in relation to greater Insall-Salvati ratio and patellofemoral congruence angle supports biomechanical mechanisms. Such work is likely to inform tissue engineering and cell regeneration approaches to improving patellar tendon morphology and outcomes in those with tendon pathology by targeting patellofemoral geometry to alter patellofemoral joint mechanisms.

## Data Availability

All data generated or analysed during this study are included in this published article [and its supplementary information files].

## Abbreviations

BMI: Body mass index
CI: Confidence interval
ICC: Intraclass correlation coefficient
MRI: Magnetic resonance imaging
